# Assessment of the effeCt of lIfestyle iNtervention plus water-soluble ciNnAMon extract On loweriNg blood glucose in pre-diabetics, a randomized, double-blind, multicenter, placebo controlled trial: study protocol for a randomized controlled trial

**DOI:** 10.1186/s13063-015-1138-7

**Published:** 2016-01-05

**Authors:** Paul Crawford, Chuong Thai, Joshua Obholz, Jeffrey Schievenin, Mark True, Sachin A. Shah, John Hallgren, Jill Clark, Danny Sharon

**Affiliations:** Nellis Family Medicine Residency, 4700 Las Vegas Blvd N, Las Vegas, NV 89191 USA; Eglin AFB Family Medicine Residency, 307 Boatner Rd, Suite 114, Eglin AFB, FL 32542 USA; Air Force Endocrinology Fellowship, Brooke Army Medical Center, 3551 Roger Brooke Dr, JBSA – Fort Sam Houston, TX 78234 USA; Pharmacy Practice, University of the Pacific, Vacaville, CA USA; 55MDOS, 2501 Capehart Road, Offutt AFB, NE 68113 USA; Nellis Clinical Investigation Program, 4700 Las Vegas Blvd N, Las Vegas, NV 89191 USA; Adolos Strategic, San Antonio, TX USA

**Keywords:** Diabetes, Cinnamon, Prevention, Pre-diabetes, Protocol, Cinnulin PF, Cinnamon extract, Impaired glucose tolerance

## Abstract

**Background:**

The World Health Organization predicts that by 2030 diabetes will be the seventh leading cause of death in the world. Multiple studies have tried to determine if cinnamon is an effective treatment for diabetes. Cinnamon extract is an insulin sensitizer, protects mesangial cells, decreases inflammatory markers, and lowers glucose, lipids, and blood pressure in patients with type 2 diabetes, so we developed a protocol to study whether ingestion of water-soluble cinnamon extract prevents progression from pre-diabetes to diabetes.

**Methods/design:**

This is a randomized, double-blind, placebo-controlled trial comparing cinnamon extract versus placebo in subjects with pre-diabetes who have committed to participate in a lifestyle change program. The trial will be conducted at five sites and will include 428 subjects who take cinnamon extract or placebo for 1 year. Follow-up for these subjects will be for a total of 2 years (nine study visits). The primary outcomes to be assessed are 1) conversion of patients from pre-diabetes to diabetes and 2) impact of water-soluble cinnamon extract on hepatic transaminases, renal function, and QT interval on electrocardiogram. Secondary outcomes include changes in HbA1c, lipids, waist circumference, weight, blood pressure, and fasting plasma glucose. The trial protocol has been approved by the Institutional Review Board of the US Air Force 59th Medical Wing, Wilford Hall Ambulatory Surgical Center (Protocol FWH20110035H). Investigator-sponsored Investigational New Drug status (114078) was granted by the US Food and Drug Administration.

**Discussion:**

This study will provide high-quality evidence of the efficacy of water-soluble cinnamon extract in conjunction with lifestyle intervention for preventing patients with pre-diabetes from converting to diabetes. Additionally, it will provide important safety information about water-soluble cinnamon extract.

**Trial registration:**

ClinicalTrials.gov Identifier: NCT01301521, 18 February 2011.

## Background

The worldwide incidence and prevalence of diabetes has reached epidemic levels—the World Health Organization predicts that by 2030 diabetes will be the seventh leading cause of death in the world [1]. Along with new drugs that target various methods for reducing blood sugar levels, more natural substances are also being investigated. One such therapy that has shown potential is cinnamon, which is purported to be a natural insulin sensitizer and has been studied and promoted worldwide.

Animal studies have suggested that cinnamon upregulates mouse adipocyte insulin receptors and may inhibit glycation end products. In previous studies, the use of cinnamon extract (CE) has been demonstrated to ameliorate intestinal insulin resistance and may be beneficial in the control of lipid metabolism [2]. Rafehi and colleagues [3] continue to study the changes in gene expression caused by Cinnulin PF, while Luo and colleagues [4] isolated three new phenolic glycosides, cinnacassosides A-C (1-3), together with 15 known compounds from the water-soluble extract of Chinese cinnamon and tested these isolates in mesangial cells to determine if cinnamon extract is useful in preventing diabetic nephropathy. They found that five unnamed compounds could inhibit over-secretion of IL-6, collagen IV, and fibronectin against high-glucose-induced mesangial cells. Additionally, in the presence of water-soluble cinnamon extract [5], the expression of genes coding glucose transporter (GLUT) increased GLUT1. Furthermore, CE decreased the expression of further genes encoding insulin-signaling pathway proteins [6].

Multiple randomized controlled trials (RCTs) of various size and quality studying cinnamon in humans with type 2 diabetes have been published with conflicting results [7–12]. Two studies showed a possible effect of cinnamon on fasting serum glucose, but hemoglobin A1c (HbA1c) levels were not examined [6, 7]. One study showed no effect on plasma glucose [9]. Two studies involving both type 1 and type 2 diabetes showed no effect of cinnamon on HbA1c [10, 11]. Cinnulin PF supplementation reduced fasting blood glucose and systolic blood pressure and increased lean body mass in an RCT of men and women with metabolic syndrome [12]. A meta-analysis of these studies in divergent populations showed no effect of cinnamon on HbA1c, glucose, or lipids [13]. In contrast, an RCT of 109 patients compared add-on treatment with 1 gram daily of cinnamon over a 3-month period to typically accepted care in a population of poorly controlled type 2 diabetics. Cinnamon lowered HbA1c an average of 0.83 %, which was similar to the effect of some prescription drugs [14]. This led to a call for more comparative research on the effectiveness of cinnamon versus prescription medications [15]. A study from 2013 found little effect of cinnamon on glucose levels in type 2 diabetes [16], and a Cochrane review that included both type 1 and type 2 diabetes found no effect of cinnamon on glucose levels [17]. However, a rigorous meta-analysis limited to evaluating the effect of cinnamon on type 2 diabetes only (10 randomized, controlled trials, *n* = 543 patients) found that “cinnamon doses of 120 mg/day to 6 g/day for 4 to 18 weeks reduced levels of fasting plasma glucose (−24.59 mg/dL; 95 % CI, −40.52 to −8.67 mg/dL), total cholesterol (−15.60 mg/dL; 95 % CI, −29.76 to −1.44 mg/dL), LDL-C (−9.42 mg/dL; 95 % CI, −17.21 to −1.63 mg/dL), and triglycerides (−29.59 mg/dL; 95 % CI, −48.27 to −10.91 mg/dL). Cinnamon also increased levels of HDL-C (1.66 mg/dL; 95 % CI, 1.09 to 2.24 mg/dL). No significant effect on HbA1c levels (−0.16 %; 95 %, CI −0.39 % to 0.02 %) was seen” [18]. Further studies since the meta-analysis found that the use of cinnamon significantly decreased both systolic and diastolic blood pressure by 5.39 mm Hg (95 % CI, −6.89 to −3.89) and 2.6 mm Hg (95 % CI, −4.53 to −0.66), respectively [19].

A survey of South Africans with both type 1 and type 2 diabetes found that more than 80 % of those surveyed were aware of the postulated positive effect of cinnamon. Thirty-six percent took cinnamon for prevention and 13 % for improved quality of life, but only 4 % took it to treat diabetes. Up to 85 % reported some degree of willingness to test the effect of cinnamon on blood glucose [20]. Residents of Sydney, Australia were also likely to try cinnamon [21]. These results support the chances that a large trial will be able to recruit adequate subjects.

Current recommendations from the American Diabetes Association regarding the treatment of pre-diabetes state that individuals with pre-diabetes should receive either intensive lifestyle intervention or metformin (Glucophage) therapy to prevent progression to diabetes [22]. In light of evidence that cinnamon extract (CE) is an insulin sensitizer, protects mesangial cells, decreases inflammatory markers, and lowers glucose, lipids, and blood pressure in patients with type 2 diabetes, we developed a protocol to study whether ingestion of water-soluble CE prevents progression from pre-diabetes to diabetes.

## Methods/design

This is a randomized, double-blind, placebo-controlled trial comparing CE versus placebo in subjects with pre-diabetes who have committed to participate in a lifestyle change program. The trial will be conducted at five sites and will include 428 subjects who take CE or placebo for 1 year. Follow-up for these subjects will be for a total of 2 years (nine study visits) and measure progression to diabetes and safety of CE. The trial protocol has been approved by the Wilford Hall Ambulatory Surgical Center Institutional Review Board (IRB) of the US Air Force 59th Medical Wing (Protocol FWH20110035H). Investigator-sponsored Investigational New Drug status (114078) was granted by the US Food and Drug Administration. Informed consent is obtained from all subjects.

## Outcomes

### Primary outcomes

The primary outcomes to be assessed are 1) conversion of patients from pre-diabetes to diabetes and 2) impact of water-soluble CE on hepatic transaminases, renal function, and QT interval on electrocardiogram.

### Secondary outcomes

Other outcomes include changes in HbA1c, lipids, waist circumference, weight, blood pressure, and fasting plasma glucose.

## Participating centers

The lead site is the 99th Medical Group at the Mike O’Callaghan Federal Medical Center, Las Vegas, Nevada. Other participating sites include the 59th Medical Wing at the San Antonio Military Medical Center, San Antonio, Texas; the 96th Medical Group at Eglin Regional Hospital, Fort Walton Beach, Florida; the 60th Medical Group at David Grant Medical Center, Vacaville, California; and the 55th Medical Group, Omaha, Nebraska.

## Recruitment

Patients will be referred by their primary care team to an approved lifestyle intervention for pre-diabetes. Interventions include Group Lifestyle Balance (GLB) instruction (a comprehensive, evidence-based, lifestyle behavior change program adapted directly from the National Institutes of Health funded Diabetes Prevention Program), nutrition classes, Better Body/Better Life, or other sessions that an investigator has certified as standard practice at that hospital. Patients attending these lifestyle interventions will be offered the opportunity to participate in this trial. Potential subjects are a combination of active duty military members and their families and retired military members and their families. Each of these Air Force medical treatment facilities treats the full range of ages and medical conditions.

The initial screening visit will include signing informed consent and HIPAA Authorization forms and initiating a record including standard demographic and contact information, name of lifestyle intervention for pre-diabetes, height, weight, waist circumference, and blood pressure. In addition, any use of statins, fibrates, niacin, or bile acid binding agents will be documented, and women of childbearing potential will have a serum pregnancy test. Subjects will be told to fast for at least 10 hours prior to their first visit.

## Inclusion criteria

Subjects included will be men and women aged 18–65 years; military beneficiaries receiving medical care at one of the participating sites; meeting diagnostic criteria for pre-diabetes [defined as fasting plasma glucose (FPG) of 100–125 mg/dl, a HbA1c of 5.7–6.4 %, or a 2-hour oral glucose tolerance test (OGTT) of 140–199 mg/dl].

## Exclusion criteria

Potential participants will be excluded from the study if they meet the following criteria: existing diagnosis of diabetes mellitus (defined as FPG ≥126 mg/dl, HbA1C ≥6.5 %, or a 2-hour OGTT ≥200 mg/dl); stage 3 or greater kidney disease [renal insufficiency defined as a glomerular filtration rate (GFR) of less than 60 ml]; celiac disease; insulinoma; Cushing’s disease; hyperthyroidism; acromegaly; pheochromocytoma; Addison’s disease; galactosemia; glycogen storage disease; hereditary fructose intolerance. Subjects already taking 1) cinnamon as a dietary supplement, 2) daily oral steroids, 3) warfarin, 4) antidiabetic medication, 5) weight loss medication, or 6) medications with narrow therapeutic indices (to include digoxin, lithium, phenytoin, and theophylline) will be excluded. Patients who are pregnant or breast feeding, known to be allergic to cinnamon or wheat (used in the placebo), and special populations will be excluded from this study.

## Randomization and blinding

Randomization was performed using a computer-generated randomization code in block sizes of 10 to ensure roughly equal sample sizes between study arms in case the trial was to be terminated early. This also allowed for an equal number of subjects to be enrolled in each arm at the individual sites. After consent, subjects will be randomized by an independent investigator not involved with subject interactions. The investigational product (IP) and placebo were manufactured in identical-looking gelatin capsules that each contain 500 mg of water-soluble cinnamon extract (Cinnulin PF) or wheat bran, respectively. Prior to the study start, IP and placebo were packaged in identical-looking opaque bottles and labeled by a team of pharmacists at the 60th Medical Group who would not dispense medication to any subject. The blinded and randomized product was shipped to each participating site for sequential enrollment of study subjects. This team then sent the blind-labeled IP and placebo to participating sites along with predesignated, randomized slips designating which bottle, A or B, a subject received. A random number generator and blocking in groups of 4 were used to ensure roughly equal sample sizes at each site. Subjects, dispensing pharmacists, research personnel, laboratory technicians, and investigators are all blinded to the study group assignments. In case of an emergency, the enrollment site’s pharmacy is to be called, and only the individual subject would be unblinded. Both IP and placebo are in identical capsules with no particular aftertaste or odor.

After enrollment, subjects randomized to consume Group A will be prescribed 2 gelatin capsules that each contain 500 mg of water-soluble cinnamon extract (Cinnulin PF) and will be instructed to take 2 capsules once a day by mouth. Subjects are to be dispensed a 3-month supply of blinded product at each site visit for a total study duration of 1 year in addition to standard of care aggressive lifestyle therapy. Group B will be prescribed 2 placebo capsules (gelatin capsule filled with wheat bran) once a day by mouth for 1 year in addition to standard of care aggressive lifestyle therapy. Each group will have the identical number of follow-up visits over 1 year with additional follow-up for 1 year after completing the study medication. Compliance with medications is to be determined with every visit using a medication adherence diary completed daily and reviewed by the study coordinators at each visit by each subject.

## Intervention

Cinnulin PF is manufactured by Integrity Nutraceuticals under Good Manufacturing Practices (GMP) and is certified by the National Sanitation Foundation (NSF). Cinnulin PF is an extract of *Cinnamomum burmannii*. A purported benefit of Cinnulin PF is that through processing almost all toxic coumarins are removed. The investigative team sent a sample of both the Cinnulin PF and placebo for toxicology analysis to Expertox Laboratory (Deer Spring, TX), and no toxin or adulterant was found in either item.

## Procedure and strategies for retention

After screening, visit 1 is to be completed within one week of the screening visit. A baseline medication list will be obtained, and a RAND 36 survey will be completed to measure quality of life. A non-investigator in the pharmacy will randomize subjects after visit 1. Subjects will be provided a pill container to assist with remembering to take their IP and a bathroom scale for weight measurement along with a “Blood Test Reminder Card,” which they will be instructed to show to their primary care team when laboratory tests are to be ordered to avoid duplication of tests. During visits 2 through 9, measurements to include weight, waist circumference, and blood pressure will be obtained; medication updates will also be obtained. At visits 5 and 9, the RAND 36 questionnaire will be repeated. Follow-up visits 2 through 9 occur 90 days after previous visit plus or minus a 10-day visit window. Subjects are given their 90-day supply of IP and encouraged to meet the 90-day mark as closely as possible. By visit 5, subjects should have completed taking all of their study medication.

### Safety assessments

All women will receive a pregnancy test at visit 1 and be instructed to use two forms of birth control while taking IP. All subjects will be questioned about adverse events (defined as any new symptom) at each visit, and all adverse events reported will be analyzed, regardless of the investigators’ assessment of causality. Research Monitors have been appointed by the IRB, and the IRB reviews adverse events annually. As part of the Investigational New Drug approval, the FDA requires safety to be assessed by complete blood cell count, liver transaminases, blood chemistry, and urinalysis. Cardiovascular events and other cardiovascular morbidity data will also be assessed by post hoc analysis. Electrocardiograms will be performed only at visits 1 and 2 to document QT intervals as part of the safety monitoring plan. Ad hoc analysis of these electrocardiograms is expected to take place after 100 subjects complete study visit 2.

Subjects are to be given a Study Medication Diary and will be instructed to bring it with them to each study visit. Subjects will be instructed to fast for at least 10 hours prior to each visit. HbA1c and a serum lipid panel (cholesterol, triglycerides, HDL, LDL) will be performed at each visit. Fasting comprehensive metabolic panel (liver function, renal function, plasma glucose tests) will be performed at visits 1 through 6 only, and a fasting plasma glucose and HbA1c will accomplished at visits 7 through 9 only. Subjects who have any of these laboratory tests done two weeks prior to a visit will not need to have these tests repeated since variability of these tests is minimal.

### Strategies for retention

Post visit 2, subjects will be seen every 90 days with a window of plus or minus 10 days. Postponement of any 6-month periodic visit is authorized for up to 6 weeks in case of a temporary condition that would affect glucose tolerance (that is, moderate to severe illness to be determined by study PI or site AI). Adherence to study medication will be assessed via review of the Study Medication Diary and recorded compliance. If a subject misplaces their Study Medication Diary, they will be asked to reproduce it from memory to the best of their ability. Subjects will be instructed to bring back all of the medication bottles, and remaining pills, if any, are to be discarded.

If at any time a subject stops taking his/her Cinnulin PF or placebo, he/she will remain in the study but is to be instructed to return any pills for documentation. During each of their follow-up contacts, research staff will offer them the opportunity to re-start their Cinnulin PF or placebo. Regardless of the amount of missed doses, all subjects are to remain in the study so that intent-to-treat analysis can be carried out and to continue with all of the study-related visits. If the subject decides to re-start their Cinnulin PF or placebo, they will resume the study at their last documented visit.

If subjects misplace or lose their study medication, they will be instructed to return to the pharmacy and will be given a new bottle from the same group of their assigned study medication. If a subject forgets to bring their study medication to a scheduled visit, they will be instructed to return it at their earliest convenience.

## Biostatistical considerations

Two null hypotheses will be tested: 1) H_0_1 - There is no difference in means of continuous dependent variables between and among treatment groups. 2) H_0_2 - Both the Cinnulin PF + Lifestyle and Placebo + Lifestyle groups have identical survival functions for the time to reach the type 2 diabetes conversion event.

## Sample size estimation and power analysis

A priori power for H_0_1 was assessed using G*Power Version 3.0.10 [23]. 214 subjects per group with 8 repeated measures (for a total sample size of 428 × 8 = 3424) will achieve a power of approximately 0.99 to detect a small effect size of 0.10 at α = 0.0004.

Post hoc analyses to evaluate differences within clinically significant time intervals will be tested with one-tailed *t*-tests for independent samples. The power analysis, using G*Power Version 3.0.10, shows that 214 subjects per group will achieve a power of approximately 0.85 to detect a small effect size of 0.20. To lessen the probability of a false negative, a β/α ratio of 1 was employed.

A priori power for H_0_2 was assessed using the method described by Glantz [24] to calculate sample size for a two-sample log-rank test to detect differences in the time to event. Based on this design, it will require a minimum of 214 subjects per group to detect a difference between a 17.5 % conversion in the Placebo + Lifestyle and an 11.5 % conversion in the Cinnulin PF + Lifestyle group at α = 0.05 (one-sided) and power of 0.80.

## Steps of analysis

The experimental design of this study is a mixed effects, randomized complete block design with repeated measures. “Subject” is a random effect, as the subjects are a sample randomly selected and randomly assigned to a treatment group. Fixed effects are treatment group and time of repeated measure, as these effects cannot be generalized to other treatments and times. Subjects will be randomly assigned to each group.

Factors to be included in the analysis are 1) Subject, 2) Treatment (Cinnulin PF + Lifestyle Management versus Placebo + Lifestyle Management), and 3) Repeated Measurements (laboratory and physical measurements obtained from each subject at fixed intervals for the duration of the study (*N* = 8)).

Outcome variables include plasma glucose, HbA1c, lipid panel (cholesterol, triglycerides, HDL, LDL), waist circumference, and conversion to type 2 diabetes (Yes/No).

Subject demographics to be collected include age, gender, and race/ethnicity (African American, European American, or Hispanic, Other).

### Descriptive statistics

Sample means and standard errors of measurement of interval variables will be calculated, and frequency distributions of nominal variables will be calculated and graphed for the total sample and for the Cinnulin PF/Lifestyle Management groups. Homogeneity of the groups will be tested with a two-tailed *t*-test and a chi-square or Fisher’s exact test for interval and nominal variables, respectively.

### Hypothesis testing

H_0_1 – This hypothesis will be tested by a mixed effects repeated measures analysis of variance (rANOVA).

H_0_2 – This hypothesis will be tested by developing Kaplan-Meier survival curves and survival functions compared with a log-rank test.

### Post hoc tests

In the event H_0_1 is rejected, contrasts will be used to investigate effects and differences within clinically significant time intervals. In the event multiple univariate tests are used to investigate effects, the Bonferroni [25] or Holm [26] method will be used to correct the level of significance for multiple comparisons.

## Discussion

This study will provide high-quality evidence of the efficacy of water-soluble CE in conjunction with lifestyle intervention for preventing patients with pre-diabetes from converting to diabetes. Additionally, it will provide important safety information about water-soluble CE.

Non-communicable diseases (NCDs) such as diabetes cause 63 % of all deaths worldwide, so strategies to mitigate and reduce the health burden of NDCs are important to improve health around the world [27]. Searching out natural compounds to prevent diabetes, verifying efficacy, and proving safety should be a priority [28].

## Trial status

Recruitment commenced on 11 June 2013, and the trial is scheduled to end in September 2016.
